# How does the globalization process improve population health? An analysis from the perspective of economic complexity

**DOI:** 10.3389/fpubh.2025.1565694

**Published:** 2025-06-11

**Authors:** Qianxue Chen, Xinzhe Zhu, Lihua Yang, Wenbo Gu

**Affiliations:** ^1^School of Business, Ningbo University, Ningbo, China; ^2^CEEC Economic and Trade Cooperation Institute, Ningbo University, Ningbo, China; ^3^Zhejiang Institute of Tianjin University, Ningbo, China

**Keywords:** globalization, population health, economic complexity, complexity outlook, mechanism

## Abstract

**Introduction:**

The comprehensive connectivity brought about by globalization and the health of local populations exhibit a contradictory relationship, which has become increasingly complex, especially since the COVID-19 pandemic. This study aims to introduce a new perspective of economic complexity to explore the complex relationship between globalization and population health at the economic level.

**Methods:**

Using an unbalanced panel dataset from 179 countries/regions between 1995 and 2021, this study investigates the mechanisms and contextual conditions linking globalization to population health from the perspective of economic complexity.

**Results:**

Our results suggest that globalization is generally associated with improved health outcomes, whereas economic globalization, when viewed in a subdimensional manner, shows a negative correlation with population health. By framing economic complexity as a proxy variable for local production capacity or industrial structure, this study offers preliminary evidence on how globalization interacts with structural economic factors to shape health outcomes. Specifically, considering the economic structure as an influence channel, economic complexity plays a mechanistic role in the relationship between globalization and health, with higher complexity outlook levels potentially strengthening the positive association.

**Discussion:**

These findings, while subject to the limitations of cross-country aggregated data, provide insights for policymakers to balance global integration with health system preparedness.

## 1 Introduction

The process of globalization profoundly influences global health in ways that are beyond the control of any single country. Individual health is extensively affected by multiple globalization factors such as public health, international trade, and politics and diplomacy, making globalization inseparable from health. The United Nations Sustainable Development Goal 3 (SDG 3: Good Health and Well-being) aims to “ensure healthy lives and promote wellbeing for all at all ages.” To achieve this goal, the World Health Organization (WHO) has promoted the Global Action Plan for Healthy Lives and Well-being for All (SDG 3 GAP), which strengthens global health cooperation and policy advocacy to help countries address health issues in the process of globalization, accelerates the attainment of health-related targets under SDG 3, and has made good progress. Taking the under-five mortality rate as an example, the United Nations data shows that as of 2022, the global under-five mortality rate had dropped to 37 deaths per 1,000 live births, representing a decrease of 51% compared with that in 2000. Among 200 countries and regions worldwide, 146 countries have achieved or are on track to achieve SDG 3.2.1, which aims to reduce the under-five mortality rate to no more than 25 deaths per 1,000 live births (1). Although globalization has provided opportunities for global health cooperation and has made some progress in promoting the realization of SDG 3, it has also introduced new challenges, particularly by exacerbating the spread of infectious diseases and leading to epidemics or pandemics. WHO Global Health Assessment data show that prior to the outbreak of the COVID-19 pandemic, the global life expectancy at birth (LEB) had increased from 66.8 years in 2000 to 73.1 years in 2019, but during the short span of 2 years from 2020 to 2022 during the COVID-19 pandemic, global LEB decreased to 71.4 years,^1^ reversing nearly a decade of gains and increasing the number of people with stress and anxiety disorders (2), severely hindering the progress toward achieving SDG 3. Therefore, understanding how the globalization process can further sustainably improve population health levels through specific mechanisms remains an important topic in the field of global health.

The conclusions of the academic community on the relationship between globalization and health are similar to the complex realities, indicating that the impact of globalization on health presents complex and contradictory outcomes (3, 4). Globalization has accelerated the global flow of medical resources, such as global medical assistance, trade in medical supplies, the transfer of medical technology, and the dissemination of advanced treatment options (5, 6), a phenomenon particularly evident in developing countries such as those in sub-Saharan Africa (SSA) (7, 8). Although globalization has improved the availability of medical technology (3), global industrialization and global trade have also indirectly caused a variety of health issues, such as the impact of climate change on diet-related health (9), the impact of greenhouse gases and other environmental pollutants on health (10), the increase in the global burden of noncommunicable diseases due to unhealthy lifestyles (11), and the mental health problems caused by global public health events like COVID-19, which affect residents’ living standards (12). In quantitative studies of the relationship between globalization and health, researchers have extensively explored how globalization affects population health through channels such as international trade (13, 14), cross-border investment (15), labor transfer (immigration) (16), digital technology (17), and inequality (18). In general, the findings of the current studies on the impacts of globalization on health remain inconclusive. Therefore, it is necessary to focus on the comprehensive factors of how more complex economic, social, cultural, and institutional changes indirectly shape health outcomes.

Economic complexity theory provides a new perspective from which to explain the relationship between globalization and health (19, 20). Economic complexity refers to the degree of complexity in the economic production structure of a country or region, reflecting its set of endogenous capacities in terms of physical capital, human capital, organizational institutions, and organizational capabilities to produce and export complex products comprehensively (21, 22). Economic complexity also encompasses the key information about the ability of an economy to generate and distribute income (23). Globalization is an important driving force for enhancing economic complexity, as it promotes international trade and the formation of cross-border industrial chains and deepens the global industrial division of labor. The division of labor determines that developed economies produce a greater variety of more complex products, while underdeveloped economies tend to produce single-category, low-technology-complexity products (24). In addition, economic complexity employs dimensionality reduction techniques on trade data, as trade data are considered an outcome indicator jointly influenced by various market-related factors and are used to assess the production capacity of globally comparable countries or regions (25). By quantifying the economic structure, economic complexity theory not only is applied to the study of economic growth and the spatial distribution of economic activities (26–28) but also has been proven to be closely related to the national social welfare level and public health (19, 29). At present, economic complexity is receiving extensive attention in the field of sustainable development, with research topics covering ecological footprints (30, 31), carbon emissions (32–34), and the energy mix (35, 36). However, the public health consequences brought about by the environmental impacts of economic complexity remains relatively understudied.

In summary, the perspective of economic complexity not only provides a theoretical basis for understanding the complex interactions between globalization and health but also offers a measurement technique to predict a country’s current and future capacities for producing various complex products, namely, the economic complexity index (ECI) and the complexity outlook index (COI). These tools help us better grasp the key mechanisms for health improvement in the process of globalization and provide policymakers with effective tools to address the related challenges. To this end, this study employs a high-dimensional panel data econometric analysis method to investigate a dataset of 179 countries/regions during the period from 1995 to 2021 with a unified framework and empirically study the relationships among the globalization process, economic complexity, and population health. This study addresses primarily the following questions: (1) Has the process of globalization effectively improved population health levels in various countries? How do different dimensions of globalization (economic, social, and political) affect population health differently? (2) From the perspective of economic complexity, what is the association among globalization, economic complexity, and health? Will higher complexity outlook level enhance the role of globalization in further improving population health levels? Answering these questions will provide a basis for national decision-makers to scientifically implement an open and inclusive sustainable population health policy.

This study contributes to the current research on the relationship between the globalization process and population health by expanding the perspective of economic complexity. First, we reexamine the binary relationship between the globalization process and population health levels based on the latest datasets. Second, we assess ECI as a potential structural channel through which the globalization process improves population health, specifically considering the possible intrinsic link between the globalization process and economic complexity. This study attempts to further expand the scope of reach on the impact of economic complexity on sustainable development. Previous studies have focused mostly on the impacts of economic complexity on energy and ecology and paid less attention to the consequences of such impacts on population health. Finally, for the first time, we investigate the impact of different levels of national COIs on the relationship between the globalization process and domestic population health levels. Unlike previous studies on economic complexity, the present study preliminarily attempts to analyze the role of economic complexity in the relationship between globalization and health.

The remainder of the paper is structured as follows. Section 2 introduces the theoretical mechanisms and research hypotheses regarding the impact of globalization on health, Section 3 presents the research methods and data, Section 4 provides the empirical results, Section 5 integrates offering actionable policy implications grounded in the empirical findings while critically addressing the study’s limitations and proposing avenues for future research.

## 2 Theoretical basis and research hypotheses

### 2.1 Globalization process and population health

The WHO Glossary defines globalization on the basis of the following two interrelated elements: the opening of borders to increasingly fast flows of goods, services, finance, people and ideas across international borders; and the changes in institutional and policy regimes at the international and national levels that facilitate or promote such flows. This definition indicates that globalization is not only reflected in the close economic and trade exchanges among countries but also accompanied by global interaction at the social and political levels. Therefore, when studying the impact of globalization on population health, it is necessary to consider economic, social, and political dimensions comprehensively.

#### 2.1.1 Economic globalization and health

Economic globalization has provided an important boost to the improvement in global health through market opening, technology diffusion, and capital flow. Especially in countries with well-established infrastructure and sufficient human capital, such as China, Vietnam, and the four East Asian tiger economies, economic globalization has significantly improved health indicators (37, 38). In addition, economic globalization has a positive impact on health indicators such as infant mortality and life expectancy in underdeveloped countries (39). However, economic globalization does not always lead to health improvements. In certain contexts, neoliberal economic policies often lead to capital inflows that exacerbate inequality and weaken the stability and equity of health systems (40, 41). Moreover, the pressure of global economic competition has limited the ability of many governments to formulate public policies, particularly reducing the amounts of financial investments of welfare states in public health (18). In Africa and Latin America, due to the asymmetry of global markets and the weak internal conditions of these countries, the health benefits of economic globalization have not been fully realized and have even exacerbated health deterioration in these regions due to global economic fluctuations (37).

#### 2.1.2 Social globalization and health

Social globalization plays a significant positive role in promoting health improvement, but the challenges it brings about cannot be ignored. On the one hand, social globalization provides new opportunities for health improvement through information flow, cultural exchange, and global connectivity. With the increase in the level of development, social globalization has gradually become an important driving force for improving health. For example, the dissemination of health knowledge and the optimization of lifestyles have been facilitated by advances in global information technology (42). The importance of social globalization is particularly prominent in addressing global health issues. Taking the COVID-19 pandemic as an example, the extensive application of information technology not only facilitated international research collaboration but also promoted resource sharing, thereby strengthening global public health cooperation (43). On the other hand, social globalization has also brought about some undeniable negative effects. First, social globalization may exacerbate the spread of cross-border infectious diseases, increasing the difficulty of global public health governance (3). Second, global technological changes and labor market liberalization have, in some cases, widened social inequality, undermining the equity and accessibility of public health systems (44). In addition, the high degree of interconnectivity of social networks has had a complex impact on individual wellbeing. Although social networks provide a platform for information sharing and psychological support, they may also amplify the uncertainty of the economic environment and personal pessimism such as stress, anxiety, and depression caused by unemployment or economic panic (45, 46), posing potential threats to mental health.

#### 2.1.3 Political globalization and health

The political dimension of globalization is reflected mainly in global governance and international cooperation, providing important guarantees for health improvement. Political globalization has elevated the priority of global health issues and promoted the coordinated efforts of the international community in areas such as health resource distribution, policy coordination, and disease prevention and control (47, 48). In countries with a higher degree of democratization, political globalization is also closely related to higher life expectancy (49, 50). Conversely, political globalization may have negative health impacts in certain circumstances. For example, Bergh and Nilsson (51) found that in countries dominated by authoritarian regimes, the impact of political globalization on health may be negative because asymmetries and additional conditions in global governance (such as patent laws and social clauses) restrict the development of health systems in developing countries, further exacerbating health inequalities (37, 41). In addition, Siiba et al. (10) examined the impact of economic sanctions on population health and health systems in Iran over the past two decades. Although the mortality indicators showed an upward trend during the sanction period, the study failed to provide strong evidence of a significant correlation between these trends and the sanctions imposed on Iran.

In summary, the impacts of globalization and its various dimensions on population health are complex. Although globalization has posed challenges in the public health field by causing a series of health inequality issues, it has provided a significant driving force for health improvements; since the acceleration of globalization in the late 20th century, human life expectancy has experienced a remarkable increase worldwide. Considering that the process of globalization is inevitable and that health inequalities are rooted in developmental gaps within countries and across the world, the transition from health inequality to health equality and establishment of a sound local medical and health system remains a gradual process (42). Therefore, we deduce the following hypothesis:

*H1:* The globalization process is associated with improved population health levels.

### 2.2 Perspective of economic complexity

Economic complexity theory aims to understand the dynamic processes of national economic development and industrial upgrading from a systems and network perspective (52). Its core premise asserts that economic growth extends beyond capital or labor accumulation; instead, a country’s economic performance depends not only on the traditional factors of production (e.g., capital, labor, natural resources), but also on how productive capacities—defined as the ability to combine technical knowledge, production experience, managerial expertise, and institutional frameworks—are organized within the economic system (53). Critically, these capacities enable economies to upgrade toward producing increasingly complex technologies, thereby shaping heterogeneous economic structures. The resulting synergy between knowledge diffusion, production structure optimization, and social welfare enhancement (29) suggests that economic complexity may mediate the impact of globalization on population health. From the perspective of economic complexity, the impact of globalization on health is constrained by the complexity level of current production knowledge and influenced by the potential for future economic development. Specifically, globalization promotes the progress of economic complexity, but its actual effect on health improvement depends on the level of economic complexity in a country or region and is further influenced by expectations for the development of its economic complexity.

#### 2.2.1 ECI as a mechanism factor

The level of ECI represents the complexity of the export products of a country or region and measures its ability to produce knowledge and apply knowledge in increasingly complex industries, reflecting an economy’s capacity to produce and export diverse and complex products (20, 23, 26). Vu (19) was the first to study how a country’s economic structure (reflected in the production and export of complex products) affects population health and demonstrated that countries producing and exporting complex (high-productivity) products enjoy better health outcomes than do those relying on less complex (low-productivity) goods, providing valuable insights into the relationships among globalization, economic structure, and health outcomes. Considering ECI as a measure of structural transformation, first, globalization enhances economic complexity by integrating economies into global value chains and promoting technological innovation and industrial diversification (54–56), thus encouraging countries to establish more advanced production capacities. Second, unlike traditional indicators such as GDP, which fail to account for production quality and diversity, ECI emphasizes the role of complex, high-value-added products in shaping economic performance and social welfare (20). For example, the transition from low-productivity sectors to high-productivity industries can promote the improvement in health status by creating employment opportunities and increasing income (57). In addition, complex economies are usually at the forefront of health-related technologies and products (58), which helps ensure local health levels. Therefore, we hypothesize the following:

*H2:* ECI playing a mechanism role in the positive relationship between globalization and population health.

#### 2.2.2 COI as an interaction factor

Moreover, the perspective of economic complexity provides a method for assessing the potential of a country or region to expand into more complex production areas based on its existing capabilities, known as the complexity outlook (COI). Unlike ECI, which reflects current production knowledge, COI indicates future economic potential (59). First, COI is used to quantify a country’s connectivity and potential in the field of complex products (60). Considering that countries with higher COI values are usually better positioned to produce complex, high-value-added products and that medical manufacturing products often require specialized and complex technical knowledge (61), this potential is crucial for determining whether a country can sustain and expand the health benefits of globalization. Second, the interaction between globalization and health is influenced by a country’s ability to seize the opportunities provided by globalization, and COI can strengthen this capacity–expansion relationship. The reason for this is that countries with high COI values are better able to leverage the opportunities brought about by globalization to expand their economic complexity and create long-term resources and innovations to improve health than are those with low COI values. Moreover, some scholars have noted that high-COI economies are usually more capable of implementing adaptive and forward-looking policies (62), including public health policies addressing health inequalities or the spread of infectious diseases, than are low-COI economies. However, other scholars argue that future economic potential may exacerbate environmental challenges while driving growth (63), thereby negatively impacting local health. Given the inverted U-shaped relationship between COI and ECI (64), this negative impact is more likely to occur in countries with advanced production structures than in those without advanced production structures. Therefore, in the context of current globalization, COI may still be a positive interaction factor, strengthening the relationship between globalization and health. Therefore, we propose the following hypothesis:

*H3:* COI is expected to positively interact the relationship between the globalization process and population health levels.

**Figure 1:**
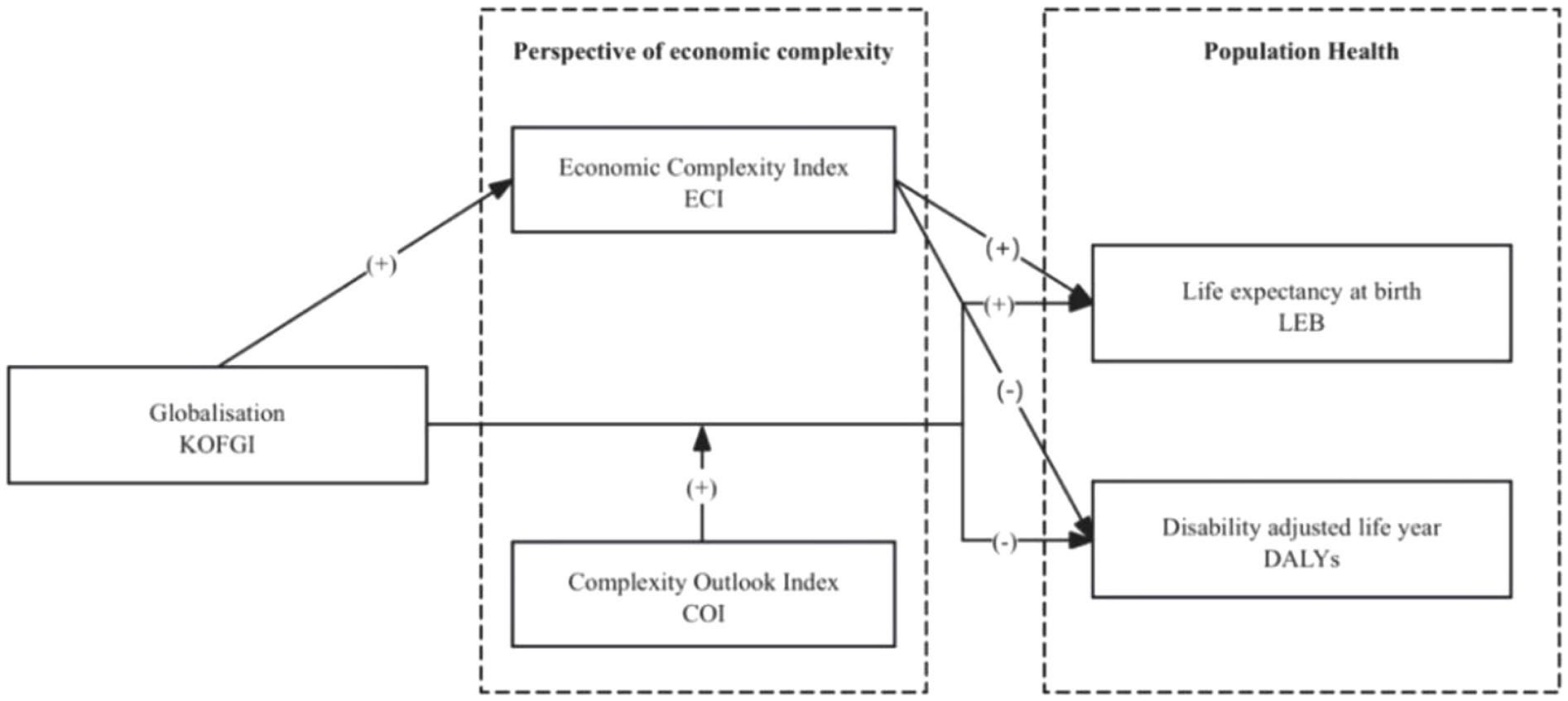
Research framework.

In summary, we draw a research framework diagram as follows (Figure 1).

## 3 Methodology and data

### 3.1 Model settings

To test the research hypotheses and support further analysis, we used three “year-country” two-way stationary panel regression models to discuss the impact of the globalization process on population health from the global perspective of economic complexity—a baseline model, a mechanism model, and a contextual interaction model, which were used to test H1, H2, and H3, respectively—to reveal the direct and indirect impacts of globalization on population health.

- Baseline model

First, we constructed a baseline regression model to test the direct impact of globalization on population health (H1). This model used panel fixed effects (FE) regression to eliminate the influence of time trends and unobservable characteristics of the sample, as shown in Equations 1, 2:


(1) $$\mathrm{LE}B_{\mathrm{ct}}=\alpha_{0}+\alpha_{1}\mathrm{KO}F_{\mathrm{ct}}+\alpha_{2}\text{Control}s_{\mathrm{ct}}+\upsilon_{t}+\mu_{c}+\varepsilon_{\mathrm{ct}}$$

(2) $$\text{InDALY}s_{\mathrm{ct}}=\alpha_{0}+\alpha_{1}\mathrm{KO}F_{\mathrm{ct}}+\alpha_{2}\text{Control}s_{\mathrm{ct}}+\upsilon_{t}+\mu_{c}+\varepsilon_{\mathrm{ct}}$$

where $\mathrm{LE}B_{\mathrm{ct}}$ and $\text{lnDALY}s_{\mathrm{ct}}$ are the logarithms of life expectancy at birth and disability adjusted life years for the country or area *c* in year *t*, respectively, which is used to characterize the health of the population; $\mathrm{KO}F_{\mathrm{ct}}$ is the level of globalization in country or area *c* in year *t*; ${\text{Controls}}_{\mathrm{ct}}$ represents the control variables; $u_{c}$ and $v_{t}$ represent country and time FE, respectively; and $\varepsilon_{\mathrm{ct}}$ is a potential random error term.

- Mechanism model

Second, to consider the structural transformation mechanism of the ECI in the impact of globalization on population health (H2), we referred to the methods of Alesina and Zhuravskaya (65), Wang et al. (66) to establish the following mechanism model (Equations 3–5) based on Equations 1, 2:


(3) $$\mathrm{EC}I_{\mathrm{ct}}=\alpha_{0}+\alpha_{1}\mathrm{KO}F_{\mathrm{ct}}+\alpha_{2}\text{Control}s_{\mathrm{ct}}+\upsilon_{t}+\mu_{c}+\varepsilon_{\mathrm{ct}}$$

(4) $$\mathrm{LE}B_{\mathrm{ct}}=\alpha_{0}+\alpha_{1}\mathrm{EC}I_{\mathrm{ct}}+\alpha_{2}\mathrm{KO}F_{\mathrm{ct}}+\alpha_{3}\text{Control}s_{\mathrm{ct}}+\upsilon_{t}+\mu_{c}+\varepsilon_{\mathrm{ct}}$$

(5) $$\begin{array}{l} \text{InDALY}s_{\mathrm{ct}}=\alpha_{0}+\alpha_{1}\mathrm{EC}I_{\mathrm{ct}}+\alpha_{2}\mathrm{KO}F_{\mathrm{ct}}+\alpha_{3}\text{Control}s_{\mathrm{ct}}+\\ \upsilon_{t}+\mu_{c}+\varepsilon_{\mathrm{ct}} \end{array}$$

where $\mathrm{EC}I_{\mathrm{ct}}$ is a proxy variable for a country’s industrial structure level measured by economic complexity.

- Interaction model

Finally, to consider the contextual situation where the future industrial complexity outlook of a country may affect the impact of globalization on population health (H3), we included an interaction term of a country’s COI and globalization level in Equations 1, 2 to obtain the following Equations 6, 7:


(6) $$\begin{array}{l} \mathrm{LE}B_{\mathrm{ct}}=\alpha_{0}+\alpha_{1}c.\mathrm{KO}F_{\mathrm{ct}}*c.\mathrm{CO}I_{\mathrm{ct}}+\alpha_{2}\mathrm{KO}F_{\mathrm{ct}}+\alpha_{3}\mathrm{CO}I_{\mathrm{ct}}+\\ \alpha_{4}\text{Control}s_{\mathrm{ct}}+\upsilon_{t}+\mu_{c}+\varepsilon_{\mathrm{ct}} \end{array}$$

(7) $$\begin{array}{l} \text{InDALY}s_{\mathrm{ct}}=\alpha_{0}+\alpha_{1}c.\mathrm{KO}F_{\mathrm{ct}}*c.\mathrm{CO}I_{\mathrm{ct}}+\alpha_{2}\mathrm{KO}F_{\mathrm{ct}}+\alpha_{3}\mathrm{CO}I_{\mathrm{ct}}+\\ \alpha_{4}\text{Control}s_{\mathrm{ct}}+\upsilon_{t}+\mu_{c}+\varepsilon_{\mathrm{ct}} \end{array}$$

where $\mathrm{CO}I_{\mathrm{ct}}$ represents a country’s complexity outlook level as the interaction variable. To avoid the effect of multicollinearity, we refer to Qiu et al. (67), which centers the core explanatory and interaction variables when generating the interaction terms.

### 3.2 Variable selection

#### 3.2.1 Dependent variable: population health

Referring to the approach of Varbanova et al. (68), we used LEB and disability-adjusted life years (DALYs) to represent population health levels, respectively.

LEB is the average number of years that a person is expected to live under the current mortality conditions and is one of the most widely used indicators for measuring population health status (68). The data used in this study were sourced from the World Bank WDI database.

DALYs are a comprehensive health indicator used to estimate the gap between the population’s health status and the “ideal” health and survival levels. DALYs are a common tool used by economists, epidemiologists, and policy experts for population health assessment (69, 70). Unlike LEB, DALYs are a measure of loss, calculated by summing the years of life lost due to premature mortality (YLLs), which represents the years of life lost due to disease-related premature death, and the years lived with disability (YLDs), which represents the years of life lost due to disability caused by disease. Research data were sourced from the Global Burden of Disease (GBD) database of the Institute for Health Metrics and Evaluation (IHME). The DALYs were log-transformed to enhance model linearity by aligning relationships with predictors and improve scale comparability across variables through right-skew compression.

#### 3.2.2 Independent variable: globalization

Following the approach in the mainstream literature (71, 72), we used the KOF globalization index (KOFGI) to measure the degree of globalization. The KOFGI encompasses various aspects of globalization in the following three dimensions: economic, social and political (73, 74). Research data were sourced from the KOF Swiss Economic Institute of the Federal Institute of Technology Zurich.

#### 3.2.3 Economic complexity methodology

Mechanism variable: Economic Complexity Index (ECI). ECI measures a country’s current level of productive knowledge and its application in increasingly complex industries. Countries with high ECI values tend to be developed economies capable of competitively producing and exporting technologically advanced products, whereas countries with low ECI values typically produce and export products with low-level technological complexity (23, 52).

Interaction variable: Complexity Outlook Index (COI). COI uses the product space to capture the correlation between the existing capabilities of the economy, thereby facilitating easier (or more difficult) diversification in related complex production areas. A higher COI value indicates that the country has a large number of complex products nearby, whose required capabilities are similar to those present in its current production, making it more likely to produce more complex products and improve its economic complexity in the future. Conversely, a lower COI value suggests that there are fewer products near the country’s existing production capabilities, making it difficult to acquire new skills and enhance its economic complexity.

The methodological framework for measuring economic complexity is comprehensively described in *The Atlas of Economic Complexity* (59). Our analysis utilizes the Atlas dataset maintained by Harvard’s Growth Lab,^2^ which provides standardized metrics of economic complexity indices and related trade data. Developed by Harvard Kennedy School, this database extends the foundational work of Product Space Theory (59). It includes annual records from 1995 onward, covering 6,000+ products across 250 countries/territories, with derived indices such as the ECI and COI. Given the methodological rigor of the Atlas dataset—including its comprehensive coverage and algorithm standardization—we focus on empirical applications rather than replicating the theoretical and computational foundations.

#### 3.2.4 Control variables

According to Huynen et al. (75), the factors influencing globalization and long-term health levels include economic development, trade, social interactions (such as migration and conflict), knowledge, and ecosystem goods and services. We used the GDP growth rate (annual percentage) to measure a country’s economic development (GDPgrows); the percentage of total imports and exports in GDP to measure a country’s trade level (Trade), with the economic freedom index as fixed trade costs (EFI); human capital costs (HumanCap), which are based on the number of years of education and returns to education, to measure the human capital stock; the population growth rate (annual percentage) to measure a country’s labor force development level (POP); and carbon dioxide emissions (kg/PPP US dollar-GDP) to measure a country’s ecological level (CO2). Except for the human capital stock data, which were sourced from Penn World Tables 10.01, all other data were sourced from the World Bank WDI database. Due to the excessive number of tables in the article and space considerations, we present the relevant descriptions of the variables in Table 1.

**Table 1:** Variable description and descriptive statistics.

Var. name			Sources	Obs.	Mean	SD	Min.	Median	Max.
Dependent variable	LEB	Life expectancy at birth	World Bank WDI database	3,854	69.7463	9.3254	40.6400	71.7940	85.4976
	lnDALYs	Disability adjusted life year	Global Burden of Disease (GBD) database of the Institute for Health Metrics and Evaluation (IHME)	3,817	10.5390	0.4264	9.6638	10.4176	12.2184
Independent variable	KOFGI	KOF Globalization Index	KOF Swiss Economic Institute of the Federal Institute of Technology Zurich	3,854	59.2268	15.4803	22.9253	58.0981	90.9294
Mechanism variable	ECI	Economic Complexity Index	Atlas Data database of the Growth Lab at Harvard University	2,998	0.0292	1.0095	−2.7784	−0.1064	2.8589
Interaction variable	COI	Complexity Outlook Index		2,998	0.0321	1.0036	−3.7092	−0.0230	2.9660
Control variables	GDPgrows	Economic development level	World Bank WDI database	3,806	0.0355	0.0522	−0.5434	0.0372	0.8683
	Trade	Trade level	World Bank WDI database	3,513	0.8533	0.5557	0.0003	0.7360	4.4262
	EFI	Economic freedom index	World Bank WDI database	3,854	59.8561	11.1976	15.5819	59.5780	90.5115
	Human Cap	Human capital level	Penn World Tables 10.01	2,936	2.5062	0.7002	1.0533	2.5991	4.3516
	POP	Labor force development level	World Bank WDI database	3,854	1.4220	1.5493	−5.2801	1.3007	19.3604
	CO_2_	Ecological level	World Bank WDI database	3,557	0.2822	0.2452	0.0366	0.2215	2.1079

### 3.3 Descriptive statistics of the sample data

In this study, a total of 179 countries or regions worldwide from 1995 to 2021 were selected as research samples. Due to significant missing values for some indicators, to improve data accuracy, we removed from the global dataset those sample countries where there were no data for core variables in the observation years, as well as statistical years with missing core variables. The statistical descriptions of the calculation results for each variable are shown in Table 1.

From Table 1, the average life expectancy at birth (LEB) is 69.7463 years (SD = 9.3254), with substantial disparities ranging from 40.6 to 85.5 years. The median LEB (71.7940 years) exceeds the mean, indicating a right-skewed distribution—consistent with most countries clustering above the average, while a subset with extremely low LEB depresses the mean. The log-transformed DALYs (lnDALYs) exhibit moderate cross-country variation (Mean = 10.5390, SD = 0.4264), suggesting relatively homogeneous disease burden patterns globally. In contrast, the KOF Globalization Index (KOFGI) shows pronounced heterogeneity (Range: 22.9253–0.9294). Both the ECI and COI are standardized metrics, where higher values denote greater productive sophistication and stronger future diversification potential. Notably, the COI’s minimum value (−3.7092) signals structural barriers to sustainable development in certain economies, reflecting limited capacity for industrial upgrading.

## 4 Results

### 4.1 Multicollinearity and correlation

Variance inflation factor (VIF) values were used to determine possible multicollinearity problems in the three models. As show in Table 2, the main effects regression model had a mean VIF of 2.08 and a maximum value of 3.95; the regression model with the ECI as an independent variable had a mean of 1.99 and a maximum value of 3.71, the mechanism model had a mean of 2.25 and a maximum value of 4.68. When constructing the interaction term, the variable was centered to reduce the influence of multicollinearity, and as a result, the interaction effect test model had a mean of 1.88 and a maximum value of 3.72. The mean VIFs of all models were generally low, and the maximum VIF values were below the threshold of 10, indicating a weak possibility of multicollinearity in this study.

**Table 2:** Multicollinearity test.

Model	Baseline model	Mechanism model		Interaction model
		ECI as a dependent variable	Full model	
Variable	VIF			
KOFGI	3.95	3.71	4.68	3.72
Human Cap	3.6	3.29	3.34	3.3
EFI	2.17	2.07	2.09	2.07
POP	1.36	1.36	1.44	1.36
CO2	1.21	1.2	1.21	1.21
Trade	1.18	1.17	1.17	1.2
GDPgrows	1.08	1.1	1.1	1.1
ECI			2.97	
c. COI*c. KOFGI				1.05
Mean	2.08	1.99	2.25	1.88

**Figure 2:**
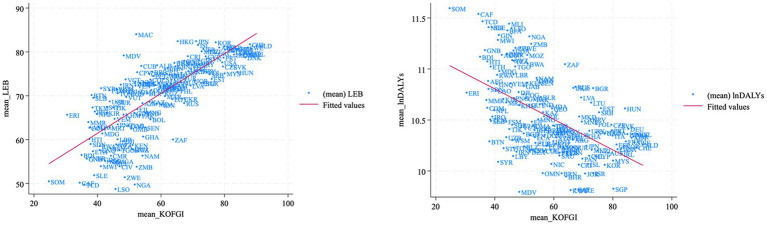
Scatterplots of the relationship between health levels and globalization.

To illustrate the approximate association between LEB and DALYs as health proxy variables and KOFGI as a globalization proxy variable, we first plotted a country scatterplot of the health level against the degree of globalization during the sample period (Figure 2). The fitted lines in Figure 2 reveal a positive correlation between mean LEB and mean KOFGI and a negative correlation between DALYs and KOFGI across countries.

Next, we performed Pearson correlation tests (Tables 3, 4), and the results revealed that LEB and KOFGI ( ρ=0.784,p<0.01 ) had a significant positive correlation and that DALYs and KOFGI ( ρ=−0.557,p<0.01 ) had a significant negative correlation, indicating a significant positive correlation between globalization and population health.

**Table 3:** Multicollinearity test: LEB-KOFGI.

Variable	LEB	KOFGI	ECI	COI	GDPgrows	Trade	EFI	|Human Cap	POP	CO_2_
LEB	1									
KOFGI	0.784***	1								
ECI	0.669***	0.778***	1							
COI	0.074***	0.056***	0.094***	1						
GDPgrows	−0.133***	−0.150***	−0.171***	0.0110	1					
Trade	0.282***	0.295***	0.246***	0.144***	0.052***	1				
EFI	0.565***	0.698***	0.540***	0.058***	−0.105***	0.355***	1			
Human Cap	0.756***	0.812***	0.724***	0.067***	−0.174***	0.301***	0.590***	1		
POP	−0.358***	−0.361***	−0.435***	0.00100	0.153***	−0.071***	−0.150***	−0.467***	1	
CO_2_	0.100***	0.036**	0.101***	0.090***	0.050***	0.051***	−0.131***	0.304***	−0.223***	1

^*^p < 0.1, ^**^p < 0.05, and ^***^p < 0.01.

**Table 4:** Pearson correlation coefficient test: DALYs-KOFGI.

Variable	lnDALYs	KOFGI	ECI	COI	GDPgrows	Trade	EFI	|Human Cap	POP	CO_2_
lnDALYs	1									
KOFGI	−0.577***	1								
ECI	−0.407***	0.778***	1							
COI	−0.043**	0.056***	0.094***	1						
GDPgrows	0.073***	−0.150***	−0.171***	0.0110	1					
Trade	−0.256***	0.295***	0.246***	0.144***	0.052***	1				
EFI	−0.430***	0.698***	0.540***	0.058***	−0.105***	0.355***	1			
Human Cap	−0.600***	0.812***	0.724***	0.067***	−0.174***	0.301***	0.590***	1		
POP	0.164***	−0.361***	−0.435***	0.00100	0.153***	−0.071***	−0.150***	−0.467***	1	
CO_2_	−0.110***	0.036**	0.101***	0.090***	0.050***	0.051***	−0.131***	0.304***	−0.223***	1

^*^p < 0.1, ^**^p < 0.05, and ^***^p < 0.01.

### 4.2 Baseline regression and robustness test

#### 4.2.1 Baseline regression

Table 5 shows the results of the empirical test for the baseline regression. Models 1 and 2 were used to investigate the impact of globalization on health without considering other factors. The results revealed that globalization significantly promoted LEB ( β=0.073,p<0.01 ) and significantly reduced DALYs ( β=−0.002,p<0.01 ). Next, we considered factors that might influence the relationship between globalization and long-term health levels and added control variables through Models 3 and 4. The results showed that the significance levels and the directions remain unchanged. Finally, Models 5 and 6 controlled for the influence of country differences and time trends on the research conclusions. Compared with those of Models 1 and 2, the results showed that the significance levels and directions remain the same, whereas the regression coefficients increased ( β=0.073→0.110 ; β=−0.002→−0.005 ).

**Table 5:** Baseline regression results.

Variable	(1)	(2)	(3)	(4)	(5)	(6)
	LEB	lnDALYs	LEB	lnDALYs	LEB	lnDALYs
KOFGI	0.073^***^	−0.002^***^	0.387^***^	−0.012^***^	0.110^***^	−0.005^***^
	(0.014)	(0.001)	(0.012)	(0.001)	(0.016)	(0.001)
GDPgrows			6.715^**^	−0.555^***^	2.790^**^	−0.226^***^
			(3.088)	(0.170)	(1.138)	(0.067)
Trade			−0.211	−0.046^***^	−0.673^***^	0.091^***^
			(0.161)	(0.010)	(0.199)	(0.014)
EFI			0.031^*^	−0.002^**^	0.025^**^	−0.001^*^
			(0.017)	(0.001)	(0.012)	(0.001)
Human Cap			3.070^***^	−0.171^***^	−0.703^*^	0.067^***^
			(0.232)	(0.016)	(0.377)	(0.023)
POP			0.050	−0.045^***^	0.108	−0.007^*^
			(0.068)	(0.005)	(0.083)	(0.004)
CO_2_			−1.044^**^	−0.013	2.940^***^	−0.314^***^
			(0.487)	(0.031)	(0.359)	(0.027)
Country_FE	YES	YES	NO	NO	YES	YES
Year_FE	YES	YES	NO	NO	YES	YES
*N*	3,854	3,817	2,685	2,685	2,685	2,685
*R* ^2^	0.9702733	0.9327937	0.7146845	0.448095	0.9751066	0.9526557
*F*	26.16403	6.883814	1009.376	292.6295	18.02709	30.22958

^*^p < 0.1, ^**^p < 0.05, and ^***^p < 0.01.

#### 4.2.2 Robustness tests

##### 4.2.2.1 Replacing the dependent variable

To avoid any measurement error that might result from the use of a single indicator, based on the discussion by Vu (19) on the relationship between economic complexity and health outcomes, the present study selected mortality indicators for different age groups as alternative measures of population health for the robustness tests. These indicators included the infant mortality rate (number of deaths per 1,000 live births, regardless of gender) MORR_inf, under-five mortality rate (probability of death before age 5 years per 1,000 live births, regardless of gender) MORR_u5, neonatal mortality rate (number of newborn deaths before reaching 28 days of age per 1,000 live births) MORR_neo, and adult mortality rates (death rates for individuals aged 15–60 years per 1,000 people, differentiated by gender) MORR_adul_f and MORR_adul_m.

According to the regression results in Table 6, after replacing the proxy variable for the dependent variable, the effects of KOFGI on the mortality rate across different age groups were all significantly negative. This finding indicates that the estimation results of the promoting effect of globalization on health remain unchanged, suggesting that the choice of dependent variable does not affect the conclusions of the present study.

**Table 6:** Robustness test results: replacing the dependent variable.

Variable	MORR_neo	MORR_inf	MORR_u5	MORR_adul_f	MORR_adul_m
KOFGI	−0.421^***^	−0.945^***^	−1.454^***^	−1.167^***^	−1.143^***^
	(0.027)	(0.068)	(0.120)	(0.270)	(0.250)
Country_FE	YES	YES	YES	YES	YES
Year_FE	YES	YES	YES	YES	YES
Controls	YES	YES	YES	YES	YES
*N*	2,685	2,685	2,685	2,671	2,671
*R* ^2^	0.9668887	0.9552018	0.9377907	0.9448407	0.9594944
*F*	63.45752	63.80862	53.15994	18.44087	7.071806

^*^p < 0.1, ^**^p < 0.05, and ^***^p < 0.01.

##### 4.2.2.2 Adding control variables

On the basis of the original control variables, to avoid the influence of potential omitted variables on the research conclusions, the present study referred to Vu (19) and included institutional quality and income inequality as control variables while also considering the impact of urban population and health expenditure on population health levels. An analysis of the regression results in Table 7 showed that after adding control variables, the direction of the estimation results of the impact of globalization on population health does not change, further validating the robustness of the conclusions.

**Table 7:** Robustness test results: adding control variables.

Variable	(1)	(2)
	LEB	lnDALYs
KOFGI	0.106^***^	−0.004^**^
	(0.031)	(0.002)
GDPgrows	−5.543^***^	0.255^***^
	(1.311)	(0.082)
Trade	0.293	0.043^**^
	(0.290)	(0.019)
EFI	0.020	0.001
	(0.017)	(0.001)
Human Cap	−0.741	0.087^*^
	(0.708)	(0.046)
POP	0.277^***^	−0.012^**^
	(0.089)	(0.006)
CO_2_	0.119	−0.093^**^
	(0.648)	(0.044)
WGI_stab	0.516^***^	−0.017
	(0.173)	(0.011)
WGI_quali	−0.308	−0.017
	(0.261)	(0.019)
Heal Exp	−0.234^***^	0.023^***^
	(0.079)	(0.005)
UPR	0.037	−0.001
	(0.030)	(0.002)
GINI	0.015	0.000
	(0.023)	(0.001)
Country_FE	YES	YES
Year_FE	YES	YES
*N*	1,092	1,092
*R* ^2^	0.9854165	0.9585516
*F*	6.269413	5.946272

^*^p < 0.1, ^**^p < 0.05, and ^***^p < 0.01.

##### 4.2.2.3 Winsorization

To eliminate possible outliers among the variables, we conducted a 1% two-sided winsorization for all variables. Table 8 reports the regression results of the dependent variable, the independent variable, and control variables after the 1% two-sided winsorization. The results showed that at the 1% significance level, the regression coefficient of KOFGI had a positive effect on population health, further supporting the conclusions of the baseline regression.

**Table 8:** Robustness test results: removing outliers.

Variable	(1)	(2)
	LEB	lnDALYs
KOFGI	0.069^***^	−0.002^**^
	(0.015)	(0.001)
Country_FE	YES	YES
Year_FE	YES	YES
Controls	YES	YES
*N*	2,395	2,394
*R* ^2^	0.9784568	0.9532576
*F*	9.184897	28.99014

^*^p < 0.1, ^**^p < 0.05, and ^***^p < 0.01.

#### 4.2.3 Endogeneity test of the instrumental variable (IV)

To address the endogeneity problem caused by reciprocal causation, following the approach of Acemoglu et al. (76), we used the wave of regional globalization as a possible exogenous source of variation in a country’s globalization process. The average level of globalization participation of the region where a specific country is located may influence the country’s level of globalization participation (77), while the wave of regional globalization does not directly affect the health level of the population in that country. Specifically, the average level of globalization in the region to which a specific country belongs^3^ was used as an IV for the country’s globalization process (78), and the two-stage least squares (2SLS) method was employed to estimate its causal impact on population health.

According to the relevance requirements for the selection of IVs, the IVs should be related to KOFGI. The first-stage regression results in Table 9 estimate the relationship between the regional globalization and the globalization of the country within that region. The IV showed a positive correlation at the 1% significance level, with an F-statistic of 45.37, which exceeds the 10% critical value for the Stock–Yogo weak identification test, indicating that the selection of this IV is valid. The second-stage regression results (IV 2SLS) showed that the exogenous component of globalization remains significantly positively correlated with health levels, specifically promoting an increase in LEB and a reduction in DALYs.

**Table 9:** Endogeneity test.

Variable	First-stage	IV (2SLS)	IV (2SLS)
	KOFGI	LEB	lnDALYs
IV_KOFGI_Region	0.235***		
	(6.74)		
KOFGI		0.101*	−0.018**
		(1.70)	(−3.88)
Country_FE	YES	YES	YES
Year_FE	YES	YES	YES
Controls	YES	YES	YES
*N*	2,675	2,675	2,675
*F*	45.37	11.99	17.48

t statistics are in parentheses; ^*^p < 0.1, ^**^p < 0.05, and ^***^p < 0.01.

### 4.3 Heterogeneity test

We are interested in whether the globalization process has improved population health levels. Most scholars have separately investigated the three dimensions of globalization:—economic, social, and political (42, 51). Therefore, we conducted regressions on the three subdimensions of KOFGI—KOFEcGI, KOFSoGI, and KOFPoGI. We found from Table 10 that the findings regarding social globalization and political globalization are consistent with those of the baseline regression, whereas some differences appear in the case of economic globalization. Although economic globalization has a certain promoting effect on increasing LEB, this effect is not significant. In addition, the advancement of economic globalization significantly increases DALYs, indicating that economic globalization has failed to make sufficient contributions to population health. Considering that diseases often spread along international trade routes (79), with the cross-border spread of the COVID-19 pandemic as the latest case, as mentioned in the Introduction, global LEB fell back to the 2012 level after the pandemic. Therefore, this result can be easily understood.

**Table 10:** Heterogeneity test: decomposing globalization.

Variable	(1)	(2)	(3)	(4)	(5)	(6)
	LEB	LEB	LEB	lnDALYs	lnDALYs	lnDALYs
KOFE	0.010			0.002^***^		
	(0.009)			(0.001)		
KOFSoGI		0.084^***^			−0.006^***^	
		(0.013)			(0.001)	
KOFPoGI			0.069^***^			−0.005^***^
			(0.009)			(0.001)
Country_FE	YES	YES	YES	YES	YES	YES
Year_FE	YES	YES	YES	YES	YES	YES
Controls	YES	YES	YES	YES	YES	YES
*N*	2,685	2,685	2,685	2,685	2,685	2,685
*R* ^2^	0.9744167	0.9748971	0.9752144	0.9522123	0.9529708	0.953733
*F*	11.44741	18.50434	17.75788	28.47125	36.67214	35.57317

^*^p < 0.1, ^**^p < 0.05, and ^***^p < 0.01.

According to a Global Burden of Disease (GBD) study, DALYs are composed of the following two parts: YLLs due to disease-related premature death and YLDs due to disability caused by disease. After decomposing DALYs, we found from Table 11 that the magnitude and direction of the regression coefficient of KOFGI on lnYLLs are basically consistent with those of the baseline regression. The impact of globalization on DALYs is reflected mainly in reducing YLLs rather than YLDs ( β=−0.000,p>0.1 ).

**Table 11:** Heterogeneity test: decomposing DALYs.

Variable	Baseline regression	Premature death	Disability
	lnDALYs	wxya	wxya
KOFGI	−0.005^***^	−0.005^***^	−0.000
	(0.001)	(0.001)	(0.000)
Country_FE	YES	YES	YES
Year_FE	YES	YES	YES
Controls	YES	YES	YES
*N*	2,685	2,685	2,685
*R* ^2^	0.9526557	0.9704147	0.9618628
*F*	30.22958	27.62366	17.85662

^*^p < 0.1, ^**^p < 0.05, and ^***^p < 0.01.

### 4.4 ECI as mechanism variable

In Section 2.2, we inferred that the complexity of a country’s production structure plays a mechanism role in the relationship between globalization and health. According to the data in Table 12, globalization has a positive impact on economic complexity ( β=0.015,p<0.01) ; economic complexity also has a positive impact on LEB ( β=0.366,p<0.05 ) but a negative impact on DALYs ( β=−0.029,p<0.01) , indicating that economic complexity significantly promotes population health. After controlling for the mechanism variable of economic complexity, the impact of globalization on population health remained consistent in terms of significance level and direction. Therefore, economic complexity serves as a mechanism variable between globalization and health, accounting for 3.67% of the mediation in the relationship between globalization and LEB and 7.8% of the mediation in the relationship between globalization and DALYs, thereby validating H2.

**Table 12:** Production structure mechanism test.

Variable	X → M	M → Y1	X, M → Y1	M → Y2	X, M → Y2
	ECI	LEB	LEB	lnDALYs	lnDALYs
KOFGI	0.015^***^		0.053^***^		−0.002^*^
	(0.002)		(0.015)		(0.001)
ECI		0.366^**^	0.269^*^	−0.029^***^	−0.026^***^
		(0.161)	(0.160)	(0.010)	(0.010)
GDPgrows	−0.424^**^	2.821^**^	2.744^**^	−0.228^***^	−0.226^***^
	(0.167)	(1.150)	(1.144)	(0.059)	(0.059)
Trade	0.104^***^	−0.631^***^	−0.775^***^	0.104^***^	0.109^***^
	(0.035)	(0.224)	(0.218)	(0.014)	(0.014)
EFI	−0.000	−0.004	−0.012	0.001	0.001
	(0.001)	(0.009)	(0.009)	(0.001)	(0.001)
Human Cap	0.254^***^	0.320	0.117	0.046^*^	0.052^**^
	(0.058)	(0.364)	(0.363)	(0.025)	(0.024)
POP	−0.029^***^	0.120^***^	0.119^***^	−0.007^***^	−0.007^***^
	(0.007)	(0.031)	(0.031)	(0.002)	(0.002)
CO_2_	0.305^***^	1.864^***^	1.960^***^	−0.239^***^	−0.242^***^
	(0.055)	(0.305)	(0.307)	(0.024)	(0.024)
Constant	−1.547^***^	70.425^***^	68.194^***^	10.332^***^	10.400^***^
	(0.192)	(1.163)	(1.388)	(0.075)	(0.087)
Country_FE	YES	YES	YES	YES	YES
Year_FE	YES	YES	YES	YES	YES
*N*	2,301	2,301	2,301	2,301	2,301
*R* ^2^	0.9606047	0.9776977	0.9778809	0.9548458	0.954929
*F*	27.88104	9.412257	10.27659	27.20713	24.78535

^*^p < 0.1, ^**^p < 0.05, and ^***^p < 0.01.

### 4.5 COI as interaction variable

To avoid the influence of multicollinearity, the core explanatory variables and interaction variables need to be centered when generating interaction terms. We incorporated COI and the centered interaction term into the regression equation and focused primarily on the estimation coefficient of the interaction term between the core explanatory variable and the interaction variable (c. COI#c. KOFGI). Table 13 reports the regression results of the interaction effects model, showing that the regression coefficient of the interaction term between globalization and COI on LEB is significantly positive ( β=0.016,p<0.01 ) and that the regression coefficient on DALYs is significantly negative ( β=−0.001,p<0.01 ). In other words, the interaction term between globalization and COI has a significant positive interaction effect on population health.

**Table 13:** Scenario impacts of complexity outlook.

Variable	(1)	(2)
	LEB	lnDALYs
c. COI # c. KOFGI	0.016^***^	−0.001^***^
	(0.004)	(0.000)
KOFGI	0.048^***^	−0.001
	(0.015)	(0.001)
COI	−0.762^**^	0.025
	(0.305)	(0.019)
GDPgrows	2.814^**^	−0.233^***^
	(1.138)	(0.058)
Trade	−0.852^***^	0.116^***^
	(0.215)	(0.014)
EFI	−0.011	0.001
	(0.009)	(0.001)
Human Cap	0.287	0.040^*^
	(0.344)	(0.023)
POP	0.117^***^	−0.007^***^
	(0.030)	(0.002)
CO_2_	2.393^***^	−0.280^***^
	(0.321)	(0.024)
Constant	67.946^***^	10.416^***^
	(1.309)	(0.082)
Country_FE	YES	YES
Year_FE	YES	YES
*N*	2,301	2,301
*R* ^2^	0.9781985	0.9556613
*F*	14.91945	28.75526

^*^p < 0.1, ^**^p < 0.05, and ^***^p < 0.01.

**Figure 3:**
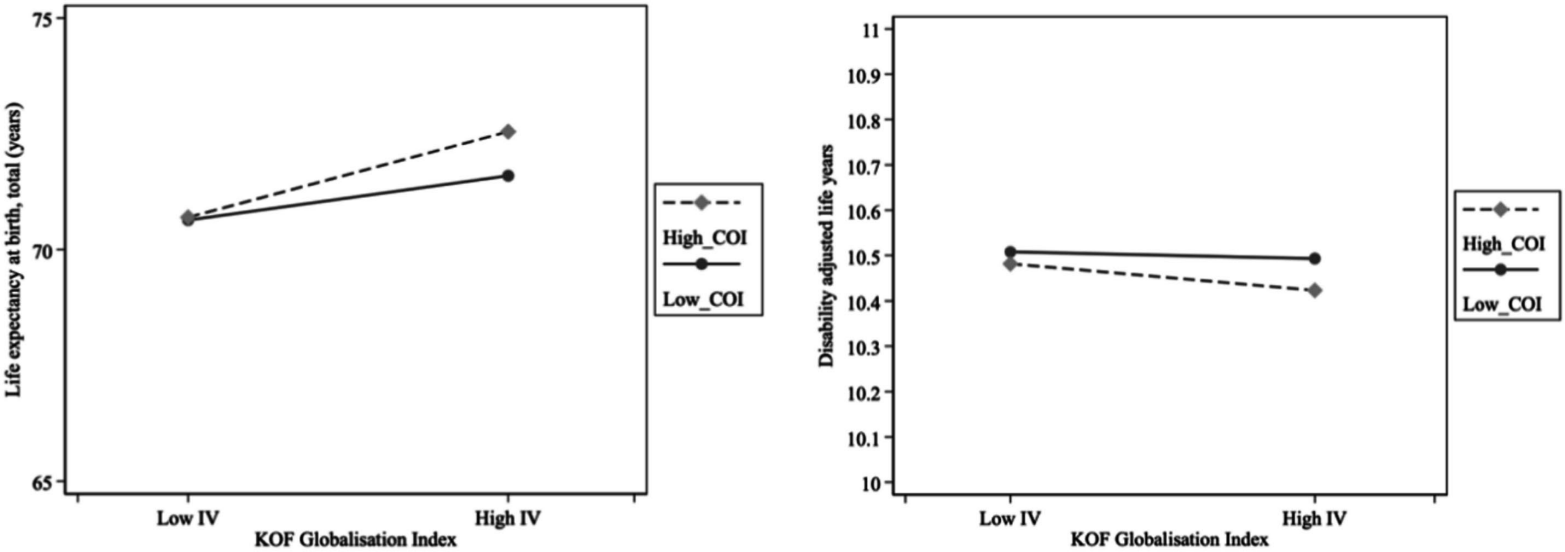
Interaction effects.

Figure 3 illustrates the interaction role of complexity outlook between globalization and health. The horizontal axis represents the difference in the level of globalization. The vertical axis in the left panel represents LEB, and the vertical axis in the right panel represents DALYs. The high level of COI is obtained by adding one standard deviation to the mean, and the low level of COI is calculated by subtracting one standard deviation from the mean. Figure 3 shows that the effects of globalization on increasing LEB and decreasing DALYs are stronger under a high level of complexity outlook than under a low level of complexity outlook.

## 5 Discussion and conclusion

### 5.1 Discussion

This study focuses on the relationship between globalization and population health, specifically examining the role of the ECI and COI in this relationship from the innovative perspective of economic complexity, in order to better understand the impacts of globalization on health and to provide valuable insights for policymakers and public health professionals.

Benchmark regressions in Table 5 indicate a statistically significant positive association between globalization and life expectancy at birth and reduces disability-adjusted life years, robust to country fixed effects and time trends. These results align with Jani et al. (42), Martens et al. (80) and Osinubi et al. (81), validating H1. Heterogeneity analyses reveal nuanced associations between globalization dimensions and population health. Contrary to earlier findings, e.g., Bergh and Nilsson (51), economic globalization exhibited a negative linkage with health during our sample period. As shown in Table 10, while its impact on life expectancy was statistically non-significant, economic globalization correlated with elevated disability-adjusted life years, primarily driven by years of life lost from premature mortality rather than years lived with disability. This paradoxical pattern may reflect COVID-19’s disruptive role during 2020–2022. Heightened economic globalization—particularly dense trade and travel networks—facilitated viral transmission (82), as well as facing a higher risk of infection and healthcare system collapse, a shock that may have contributed to the negative health effects of economic globalization while overshadowing its long-term positive effects. Conversely, political and social globalization demonstrated stronger positive linkages with health outcomes. Political integration through multilateral agreements mitigated economic globalization’s risks (83), while social globalization enhanced non-market resource allocation. For example, international organizations such as WHO coordinate vaccine development and push prevention and control guidelines during outbreaks (84), and the transnational flow of information through social media can raise public health awareness (85).

On the other hand, our mechanism analysis systematically examines ECI as a potential structural channel linking globalization to population health. As evidenced in Table 12, globalization shows a positive association with economic complexity, while economic complexity is positively associated with life expectancy and negatively associated with disability-adjusted life years. These patterns suggest that economic complexity partially mediates the globalization-health relationship, corroborating Cornia (37) proposition that the impact of globalization on health and the economy depends on a country’s internal and external conditions, thereby supporting Hypothesis 2. In addition, by introducing economic complexity into the mediation model between globalization and health, we extend Vu (19) theoretical application of economic complexity in health research, providing a theoretical basis for understanding how globalization improves health through the complexity of industrial structures. Different from the ECI, which is a static measure of countries’ existing production complexity, the COI focuses more on the strategic foresight of countries’ future product positioning (63). The results of the empirical analysis of the interaction model show that COI has a significant positive moderating effect on the country’s health gains from the globalization process. The implication is that, as Gomez-Gonzalez et al. (62) point out, high COI economies demonstrate greater strategic foresight to translate the health dividends of globalization more effectively by developing adaptive globalization-health synergistic development strategies based on their own socioeconomic conditions.

### 5.2 Conclusion

This study conducts a high-dimensional econometric analysis of a panel dataset of 179 countries or regions between 1995 and 2021 to investigate the relationship among the globalization process, economic complexity, and population health, particularly examining the roles of ECI and COI in this relationship from the perspective of economic complexity. Specifically, the study tests the direct effects of KOFGI on LEB and DALYs, as well as the roles of ECI and COI as mechanism and interaction variables, and the following conclusion was obtained:

1. Globalization-Health Associations. The KOF Globalization Index demonstrates significant positive associations with life expectancy at birth (LEB: *β* = 0.073, *p* < 0.01) and negative associations with disability-adjusted life years (DALYs: *β* = −0.002, *p* < 0.01), robust to country-level heterogeneity and temporal trends. Robustness checks-including alternative health metrics, control variable expansion, tail truncation, and instrumental variable approaches-confirm result stability (*p* < 0.05 across specifications). Economic globalization exhibits paradoxical patterns: insignificant for LEB (*p* > 0.10) but positively associated with DALYs through premature mortality (YLLs: *β* = 0.11, *p* < 0.05).
2. Perspective of economic complexity. Globalization positively correlates with ECI (*β* = 0.015, *p* < 0.01), which subsequently associates with higher LEB (*β* = 0.366, *p* < 0.05) and lower DALYs (*β* = −0.029, *p* < 0.01). Mechanism analyses indicate ECI explains approximately 3.67% of the mediation in the relationship between globalization and LEB and 7.8% of the mediation in the relationship between globalization and DALYs. The globalization-COI interaction term shows synergistic effects: positive for LEB (*β* = 0.016, *p* < 0.01) and negative for DALYs (*β* = −0.001, *p* < 0.01). Nations with higher COI levels exhibit amplified health improvements from globalization, suggesting COI functions as a complementary enhancer rather than substitute in this relationship.

The globalization-population health nexus exhibits intricate multidimensionality, requiring governments to implement context-specific, stratified development strategies. In accordance with the empirical evidence, we advance the following evidence-based policy recommendations:

Firstly, in order to optimize health outcomes in the context of globalization, policymakers should take into account the characteristics of the different dimensions of globalization and adopt a multidimensional governance framework that synergistically addresses both opportunities and risks. Social globalization should actively promote knowledge diffusion and cultural inclusion through enhanced international health data exchanges and cross-border medical technology flows. Concurrently, political globalization requires strengthening institutional mechanisms for global health governance, particularly through international organizations that facilitate public health collaboration, accelerate SDG3 achievement, and ensure equitable distribution of health benefits. Economic globalization demands careful mitigation of short-term health inequities through trade agreement safeguards and restrictions on harmful industry transfers, while maintaining its long-term development advantages.

Secondly, in terms of specific implementation paths, governments should promote the globalization strategy in a layered manner according to the differences in their economic structures. Countries with higher economic complexity should give full play to the technology diffusion effect and enhance the health governance capacity of low- and middle-income countries by exporting affordable healthcare programs; Economies with medium complexity need to focus on technological innovation and achievement transformation mechanisms for high value-added medical industries; Countries with a single economic structure should prioritize the improvement of basic medical facilities and cultivate endogenous development momentum through the selective introduction of foreign-funded technologies.

Finally, governments should take the enhancement of economic sophistication as a core policy lever and implement differentiated economic structure upgrading strategies to magnify the health dividends of globalization. Countries with complex industrial bases should strengthen the embeddedness of their global production networks, promote the extension of their advantaged industries into high-end medical fields, and accelerate the transformation of the health benefits of technological spillovers through institutional innovation; Countries with a weak industrial base need to balance technology importation and independent innovation, and cultivate local production capacity through open cooperation.

### 5.3 Limitations and prospects

This study still has several methodological and data limitations. First, while employing rigorous two-way fixed effects panel regression models, macroeconomic-level analyses cannot fully address endogeneity, particularly due to potential unobserved confounders. Second, key variable measurements rely on composite indices whose methodological differences may compromise cross-country comparability, with data limitations (especially in low-income countries) potentially introducing selection bias. In addition, the findings at the national level cannot be directly extrapolated to regional or individual contexts, which may lead to ecological fallacy. The heterogeneous impact of globalization on the mechanistic effects of different social groups and their various sub-dimensions remains under-discussed and further breached through subsequent research.

## Data Availability

The raw data supporting the conclusions of this article will be made available by the authors without undue reservation.

## Footnotes

^1^WHO Global Health Observatory: https://www.who.int/data/gho/data/themes/mortality-and-global-health-estimates/ghe-life-expectancy-and-healthy-life-expectancy (Accessed October 1, 2024).

^2^Growth Lab at Harvard University. “The Atlas of Economic Complexity.” Web application. Harvard Kennedy School, 2024. https://atlas.hks.harvard.edu (Access October 1, 2024).

^3^Standard country or area codes for statistical use (M49): https://unstats.un.org/unsd/methodology/m49/ (Accessed October 15, 2024).
